# Ultrastructural and Physicochemical Characterization of a Non-Crosslinked Type 1 Bovine Derived Collagen Membrane

**DOI:** 10.3390/polym13234135

**Published:** 2021-11-26

**Authors:** Igor da Silva Brum, Carlos Nelson Elias, Ana Lucia Rosa Nascimento, Cherley Borba Vieira de Andrade, Ronaldo Sergio de Biasi, Jorge José de Carvalho

**Affiliations:** 1 Implantology Department, State University of Rio de Janeiro, Rio de Janeiro 20550-900, Brazil; 2 Materials Science Department, Instituto Militar de Engenharia, Rio de Janeiro 22290-270, Brazil; elias@ime.eb.br (C.N.E.); rsbiasi@ime.eb.br (R.S.d.B.); 3 Laboratory of Ultrastructure and Tissue Biology, Department of Histology and Embryology, State University of Rio de Janeiro, Rio de Janeiro 20550-900, Brazil; alrosa22@hotmail.com (A.L.R.N.); cherley.andrade@uerj.br (C.B.V.d.A.); jjcarv@gmail.com (J.J.d.C.)

**Keywords:** type 1 collagen, roughness, cell matrix, wettability, DSC

## Abstract

In this work, in vitro testing was used to study the properties of non-crosslinked type 1 bovine derived collagen membranes used in bone regeneration surgery. Collagen membranes were prepared, their surface roughness was quantified by interferometry, their morphology was observed by scanning electron microscopy (SEM) and transmission electron microscopy (TEM), their wettability was measured by the contact angle technique, their mechanical properties were investigated by tensile testing, their phase transformation temperatures were measured by Differential Scanning Calorimetry (DSC), and their biocompatibility was evaluated by immunological testing. The calorimetry tests showed that the membrane is formed only by type 1 collagen. The SEM observations showed that the morphology consists of layers of highly organized collagen fibers and patterns of striated fibrils typical of type 1 collagen. The small contact angle showed that the membrane is hydrophilic, with the possibility of rapid absorption of body fluids. The tensile tests showed that the membrane has enough elasticity, ductility, and mechanical strength for use in tissue regeneration. With the immunostaining technique, it was possible to confirm the membrane biocompatibility.

## 1. Introduction

Absorbable and non-absorbable natural membranes have been used in maxillofacial surgery [1]. Non-absorbable membranes require a second surgery for removal, which increases the risk of bacterial colonization and inhibition of bone regeneration. Therefore, absorbable collagen membranes are best for tissue regeneration. The absorption time can be managed in the processing stage by providing a certain degree of crosslinking that is controlled by addition of hexamethylene diisocyanate, diphenyl phosphorylazide, or glutaraldehyde, combined with UV radiation [2].

Collagen is the most common extracellular protein matrix in the human body [3]. Among the sources of collagen for biomaterial applications, tendons and pericardium are widely used to make biomaterials, especially membranes [4]. In dentistry, collagen membranes are used to mechanically isolate defects and prevent the proliferation of epithelial cells and connective tissue in regenerated areas [5,6]. In medicine, they can be used in surgery of the skull, spine, and knee.

Collagen membranes may be crosslinked or non-crosslinked. Collagen fiber organization is the most efficient way to improve the efficiency of non-crosslinked membranes [7]. A series of analyses can be used to investigate the properties of non-crosslinked collagen membranes and their biological effects [8]. The membrane should have high wettability (>7.1 s) and also high elasticity (>14 MPa) to facilitate handling during surgery [9].

Collagen membranes are often used to minimize reactions to foreign bodies involving macrophages [10]. The literature shows that macrophages play a fundamental role in the bone remodeling process. They are attracted to pro-inflammatory and anti-inflammatory phenotypes [11]. Due to their high capacity of controlling the balance of pro-inflammatory and anti-inflammatory microenvironments, the results depend on the control of the immune system. Low-resorptive, non-crosslinked collagen membranes modulate the immune system and promote guided bone regeneration [12].

Data from immunohistochemical analyses of the reaction of monoclonal antibodies of collagen type I and V and pro-collagen type III indicate that fibroblasts are the main cells involved in collagen production [13]. Therefore, having a collagen membrane that is compatible with fibroblasts favors tissue regeneration in the surgical wound [14].

SEM and TEM are widely used to investigate the morphology of structures. In the case of collagen membranes, SEM can be used to determine the number of collagen layers and their thickness, as well as the existence of substances unrelated to collagen. Using TEM, one can determine the characteristic pattern of the structure, which, in type 1 collagen membranes, consists of well-defined fibers [15].

Other tests can be used to characterize type 1 collagen membranes, such as differential scanning calorimetry (DSC), that determines their s thermal stability [16], the wettability test, that determines if the sample has a satisfactory wettability [17], and the immunohistochemical test, that is used to determine if the material contains undesirable phases [18].

The objective of the present work was to develop a method to characterize samples of non-crosslinked type 1 experimental collagen membranes made from bovine tendon and analyze their biological properties. We tested the mechanical properties, surface roughness, wettability, phase transformation, and biocompatibility of a membrane made with bovine type I collagen.

## 2. Materials and Methods

The material characterization techniques can be used to predict the performance of biomaterials, thus reducing number of in vivo testings.

In the present work, samples of an experimental non-crosslinked bovine collagen membrane named Green Membrane^®^ 2 mm thickness and porosity ranging from 0.31 mm to 0.75 mm were used. The characterization of the membrane was carried out using tensile testing, SEM, TEM, DSC, interferometry, contact angle measurement, and immunohistochemistry.

The collagen membranes were prepared in the laboratory of Regener Biomaterials (Curitiba, Brazil) from non-crosslinked type I collagen fibers from bovine Achilles tendons, for an expected membrane reabsorption time of up to 120 days. The collagen underwent purification and processing procedures, including the use of sodium hydroxide to inactivate pathogens, according to Brazilian and international standards for handling and supplying animal tissues.

### 2.1. Surface Morphology Analysis

The most important structural morphology of membrane for bone regeneration is the cellular structure. Typically, collagen membrane morphology characterization is performed by SEM, interferometry, MRI (magnetic resonance imaging), small-angle XRD (X-ray diffraction), and AFM (atomic force microscopy). In the present work, the membrane morphology was characterized by SEM.

### 2.2. Scanning Electron Microscopy and Transmission Electron Microscopy

Gold-coated membrane surfaces were analyzed using a Field Emission GUN Quanta 250 FEG (FEI Company, Oregon, USA). A 5000 magnification was used to investigate the homogeneity, a 15,000 magnification to observe cell clusters, and a 20,000 magnification to identify specific cell types.

For the SEM analysis, the fixation procedure started with osmium tetroxide and potassium ferrocyanide (1.0 wt%, 0.8 wt%, respectively) with a cacodylate buffer (0.1 M, pH 7.4) incubation for 1 h in the dark, followed by three sodium cacodylate buffer rinses in distilled water (0.2 M, pH 7.4) for 1 h. This was followed by sequential ethanol grades (25–100 vol%) rinse for specimen dehydration. The slices were immersed in hexamethyl disilazane for 10 min before placing in an evaporation chamber for drying. Specimen mount on aluminum stubs was achieved using colloidal silver adhesive (Electron Microscopy Sciences, Peabody, MA, USA). The specimens were coated with gold film by sputtering (Cool Sputter Coater—SCD 005, Bal-Tec, Berlin, Germany).

Thin membrane sections were analyzed using a JEOL JEM-1011 transmission electron microscope (JEOL, Ltd., Akishima, Tokyo, Japan), operatin at 60 kV. Digital micrographs were captured using an ORIUS CCD digital camera (Gatan, Inc., Pleasanton, CA, USA) at 8000×, 10,000× and 25,000× magnification.

The preparation of the samples for TEM analysis was the following:

(a) fixation in 2.5 wt% glutaraldehyde diluted in 0.1 M cacodylate buffer solution (overnight);
(b) washing in 3 baths in cacodylate buffer solution (0.1 M), for 15 min each bath;
(c) dehydration in 30 vol% acetone bath (15 min), 50 vol% acetone, 70 vol% acetone (15 min), 90 vol% acetone (15 min), 100 vol% acetone (15 min), and 100 vol% acetone (15 min);
(d) infiltration in acetone + epon mixture (2:1) for 2 h; acetone + epon (1:1) for 2 h; acetone + epon mixture (1:2) for 2 h, infiltration in pure Epon (overnight);
(e) inclusion in Epon and polymerization between 48 and 72 h at 60 °C;
(f) plate cuts with a thickness of 1 micrometer and staining with toluidine blue;
(g) cutting with ultramicrotome to obtain 70 nm slides, which were collected on 300 mesh copper grids;
(h) contrasting of the slides with uranyl acetate (for 20–30 min); and
(i) TEM observation.

### 2.3. Surface Roughness and Wettability

The membrane surface roughness was investigated using a non-destructive optical interferometer NewView 7100 (Zygo Corporation, Middlefield, CT, USA) to measure the roughness parameters Ra, Rsk, Rms, Rku, PV, Rpk, Rk, and R3z. The meaning, definitions and mathematical equations to calculate the terms are available in the ISO 4287 technical standard (ISO 4287:1997 Geometrical Product Specifications (GPS)—Surface texture: Profile method—Terms, definitions, and surface texture parameters). A 3D non-destructive optical interferometer roughness measurement was used because it provides a better understanding of the surface than measurements with a 2D profilometer. The interferometry technique yields a 3D image of the measurement region. Suppose, for example, that a 2D profile shows a pit on the surface. When the 3D surface map is examined, it may reveal that the assumed pit is actually a valley and has more bearing on the function of the surface than a discrete pit.

The parameters used for 3D surface roughness measurement were the following: camera Canon, Tokyo, Japan, mode 640 × 480 72 Hz, image zoom 0.75×, scan length 300 µm, average filter, spike height higher than 3.0 µm were removed, and measurement area 0.141 × 0.188 mm. A robust Gaussian filter based on the maximum likelihood estimation was used to suppress the influence of the outliers.

The overall accuracy of the measurement results can be influenced by environmental conditions, particularly draughts, vibration and the rate of change of the ambient temperature. The instrument was far from air conditioning vents and laminar flow hoods. Both acoustic and ground based vibrations were avoided. The instrument has a compressed air anti-vibration system.

The surface wettability was determined by measuring the contact angle with a goniometer First Ten Angstroms FTA-100 (First Ten Angstroms Co., Portsmouth, VA, USA). The contact angles were determined by averaging the values obtained at five different areas on the three samples surfaces using NaCl 0.9% solution.

The wettability was quantified by the sessile drop measurement methodology, that is, by the contact angle measurement method. This methodology is the most used. For the measurement a drop of distilled water was placed on the surface of the membrane and an image of the drop was obtained. The static contact angle was defined by fitting the Young-Laplace equation around the drop.

### 2.4. Tensile Test

A satisfactory mechanical strength is essential when membranes are used in maxillofacial surgery. Membrane rupture is a potential cause of complications and loss of effectiveness. In the present work, we tested the mechanical properties of experimental bovine type I collagen using tensile testing and compared it with literature data on other collagen membranes [1].

Tensile tests provide information on the strength and ductility of materials under uniaxial tensile stress. They were performed using a Universal testing machine, EMIC DL10000 (Emic, Parana, Brazil), according to ASTM D638-14 (Standard Test Method for Tensile Properties of Plastics).

Sheet specimens were cut conforming to the dimensions described in technical standard ASTM D638-14 (length overall = 60 mm, distance between clamps = 30 mm, length of narrow section = 20 mm, gage length = 7.33 mm, and thickness = 0.31 mm).

**Figure 1:**
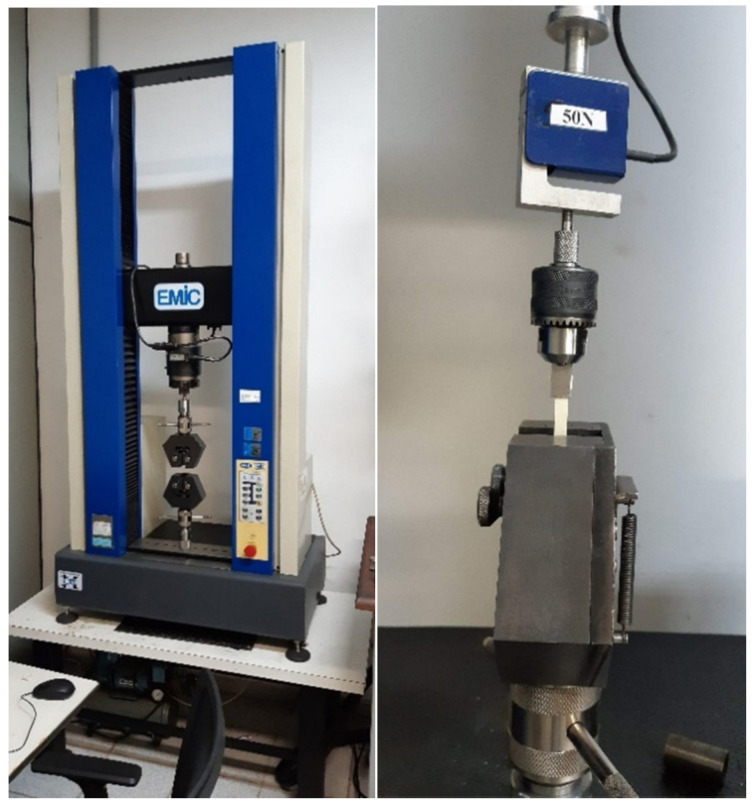
Tensile testing setup.

Before the mechanical tests, the surface of the samples was inspected to ensure that it was free of visible flaws, scratches, and imperfections. Tensile tests were performed at room temperature with a cross head speed of 10 mm/min. A high-definition force transducer of 50 N was used (Figure 1). Extreme care was taken to ensure that the test specimen be inserted and clamped so that the long axis of the test specimen coincided with the direction of pull through the center line of the grip assembly. Three parameters were measured:

(1) Maximal load (N) at the extreme loading point.
(2) Tensile strength (maximum stress, MPa) calculated as the maximal load divided by the cross-sectional area of each specimen.
(3) Maximal strain (%) calculated at the point of maximal load.

The tensile strength was calculated dividing the maximum load sustained by the specimen in newtons by the average original cross-sectional area in the gage length segment of the specimen in square meters. The percent elongation was calculated at break by reading the extension (change in gage length) at the point of specimen rupture. The Young modulus was calculated from the slope of the curve.

### 2.5. Measurement of Thermodynamic Properties Using DSC

The thermal properties of the collagen membrane were investigated using a Shimadzu DSC60 DSC calorimeter (Shimadzu, Kyoto, Japan). The DSC thermodynamic technique quantifies the variation of the thermal energy absorbed by the material during heating or cooling. During the test, two bodies are used, the sample and a reference, which are kept at the same temperature during the experiment. The difference in the amount of heat required to increase the temperature of the sample and the temperature of the reference is measured. The reference sample has a heat capacity defined over the range of temperatures being scanned. Analyzing the data, the temperatures and the energies of the phase changes and transitions can be determined.

The samples (∼7.0 mg each) were placed in aluminum pans. The pans were tightly sealed, weighed, and DSC scans were performed with a scanning rate of 5 °C/min between 25 °C and 150 °C. The tests were performed under nitrogen atmosphere, with a gas flow of 30 mL/min. The reference was an empty aluminum pan.

The thermal measurements yielded the temperature and energy for membrane denaturation. The phenomenon of denaturation is distinct from degradation. Denaturation is the rupture of interchain hydrogen bonds that leads to formation of an amorphous material. The temperature at the beginning of denaturation (*T*_onset_), the temperature at the end of denaturation (*T*_endset_), the peak temperature of denaturation at maximum heat absorption (*T*_p_), the change of enthalpy (Δ*H*), and the width at half-peak height (Δ*T*_1/2_) were determined from the DSC curve. The peak temperature of denaturation is the temperature at which collagen structure unfolding takes place. The thermal denaturation of collagen membrane was characterized by its enthalpy (Δ*H*_d_) and denaturation temperature (*T*_d_). The change of enthalpy (Δ*H*) corresponds to the energy absorbed by the tissue during helix-coil transformation of the collagen.

### 2.6. Immunohistochemical Analysis (In Vitro)

For the preparation and analysis of the samples, the sequence of steps was the following:

(a) deparaffinize the sections in 3 xylol baths (5 min each bath);
(b) hydrate the sections (100%, 90%, 70% ethanol, distilled water—5 min each bath);
(c) incubate the sections in 3% hydrogen peroxide diluted in distilled water, for 15 min, protected from light to inhibit endogenous peroxidase;
(d) wash in 3 baths of TBS (Tris Buffered Saline) pH 7.4 buffer (5 min each bath);
(e) perform antigen retrieval in Tris/EDTA (ethylenediaminetetraacetic acid) buffer pH 9.0 at 95 °C, for 20 min;
(f) cool and wash in 3 baths of TBS buffer pH 7.4 (5 min each bath); and
(g) block non-specific sites with 3% TBS/BSA for 20 min.

The final steps were: incubate with the primary anti-Collagen I antibody (Santa Cruz), diluted in TBS/BSA at 1% (1:200), overnight, in a refrigerator (4 °C), in a humid chamber; wash in 3 baths of TBS buffer pH 7.4 (5 min each bath); incubate with biotinylated secondary antibody (VECTASTAIN^®^ Universal Quick HRP Kit) for 30 min at room temperature; wash in 3 baths of TBS buffer pH 7.4 (5 min each bath); incubate with streptavidin VECTASTAIN^®^ Universal Quick HRP Kit) for 15 min at room temperature; wash in PBS buffer pH 7.2; develop with DAB (diaminobenzidine); stain with hematoxylin; dehydrate (distilled water, 70%, 90% and 100% ethanol—5 min each bath); and clarify in 3 xylol baths (5 min each bath) and assemble with Entellan.

## 3. Results

### 3.1. Surface Morphology

**Figure 2:**
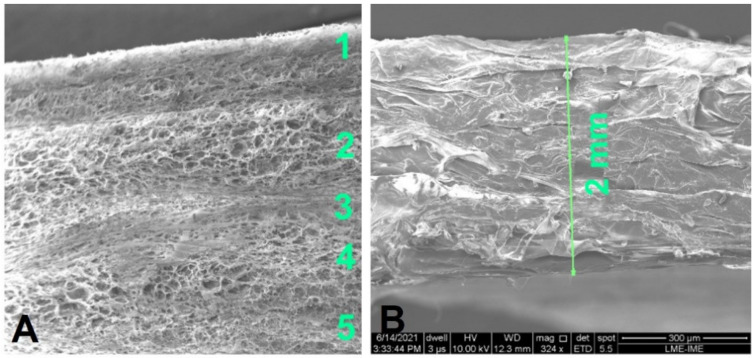
Type I collagen membrane morphology. One can see 5 layers of collagen fibers (**A**) and marginal sealing (**B**). The characteristics of the membrane ensure good elasticity, wettability, and permeability.

Figure 2 shows that the membrane has 5 layers of collagen fibers (Figure 2A), well defined and with sealed edges to ensure stability at the insertion site (Figure 2B). The aforementioned characteristics allow the membrane to exhibit slow resorption.

**Figure 3:**
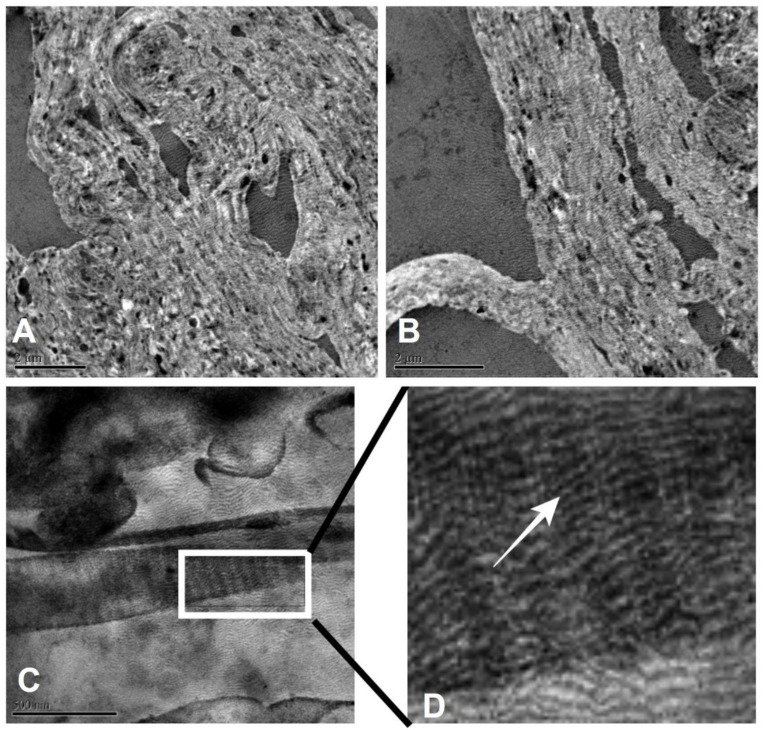
Type 1 collagen fibril microstructure observed by transmission electron microscopy. One can see the characteristic fibrillar pattern of type 1 collagen. Micrograph magnifications were 8000× (**A**), 10,000× (**B**), 8000× (**C**), and 25,000× (**D**).

TEM can be used to characterize the structures of materials, especially to visualize tissues. The use of the backscatter electron (BSE) technique provides the contrast of regions with different chemical composition and morphology (Figure 3). Since the regions with elements of high atomic mass backscatter the incident electron beam more strongly than the regions with elements with low atomic mass, the regions with high concentration of heavy elements appear brighter than the other regions.

### 3.2. Roughness and Wettability

Since it is not possible to characterize a surface completely with a single parameter, a combination of parameters is normally used. Roughness average (R_a_), the most common1y used roughness parameter, is defined as the mean height of the roughness profile. There are doubts as to whether the variations in the heights of surface irregularities are more important than the distance between them, or what the best combination of these factors for membrane performance is. In the present work, six roughness parameters have been measured. The definitions of the main roughness parameter are:

- Ra (average roughness): Ra is average value of the amplitudes in relation to a reference line. This parameter quantifies the average vertical distance between the five highest peaks and five major valleys.
- R_sk_: asymmetry factor (skewness).
- R_ms_ (root mean square roughness): R_ms_ is the root-mean-square deviation of all points from a plane fit to the test part surface.
  R_ku_: flattening profile (kurtosis).
- PV: the height between the lowest and the highest point on the test area.
- Rpk: maximum height of the highest peak of surface roughness, situated above the midline.
- Rk: the core height of the profile along the Y-axis of the BAC curve generated by placing a 40% line on the curve at the minimum slope point and extending the lines to the 0% and 100% points.
- R3z (average roughness of the third peak). In each module, the distances between the third highest peak and the third deepest valley are plotted. R3z is the height of this peak plus the depth of the valley.

**Figure 4:**
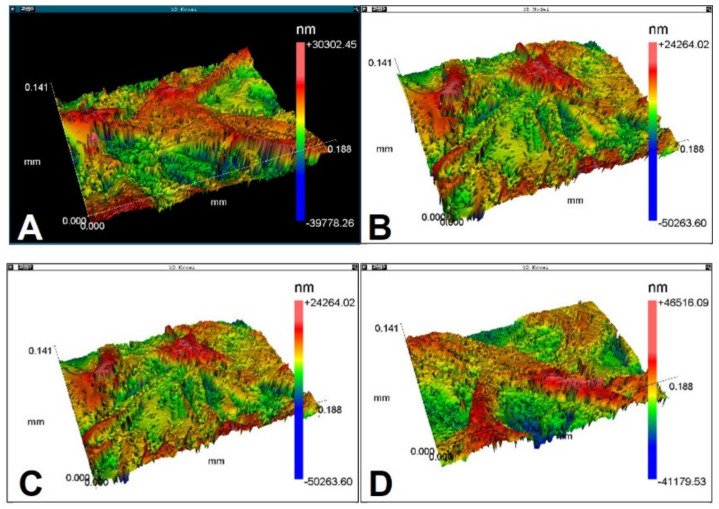
Three-dimensional morphology of sample roughness, obtained by interferometry, displaying a favorable roughness. (**A**,**B**) The side that is in contact with mucosa; (**C**,**D**) the opposite side.

With the technique of interferometry, it was possible to analyze the surface morphology and quantify the roughness parameters of the samples. Membrane roughness influences biological performance. Membranes must have a roughness with characteristics that allow interaction with proteins and cells. Table 1 shows the membrane surface roughness parameters that were measured. Figure 4 shows a membrane surface morphology observed by interferometry. The membrane surface has an irregular shape, with valleys and peaks.

**Figure 5:**
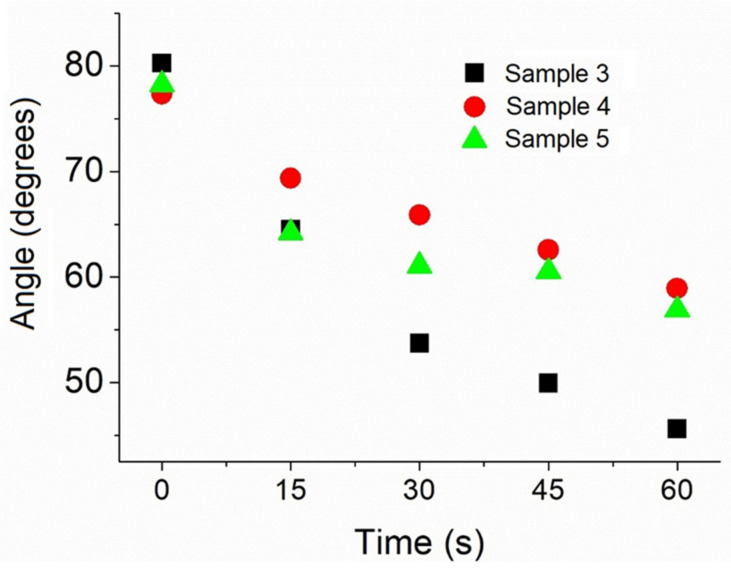
NaCl drop contact angle with the surface of samples 3, 4, and 5 as a function of time after drop deposition. The contact angle with the surface of samples 1 and 2 was measured only at *t* = 0.

Membrane wettability was quantified depositing a 1.0 mL drop of 0.9% NaCl on the surface. The larger the membrane wettability, the greater the number of cells adhered in the regeneration process. The morphology of the membrane shown in Figure 3 facilitates the infiltration of fluids through the collagen fibers by capillary action. Table 2 and Figure 5 show the variation of the contact angle with time after drop deposition. The fact that the contact angle decreases significantly with time indicates that the membrane is hydrophilic. Table 3 shows the drop parameters (base diameter, base area, height, sessile volume, and sessile surface area) as functions of time after deposition.

Typically, researchers only measure the roughness parameter Ra. In the present work, 8 parameters were measured (see Section 2.3 and Table 1). Considering that the samples had similar roughness parameters, it was not possible to analyze the influence of roughness on wettability (Table 2).

### 3.3. Tensile Tests

**Figure 6:**
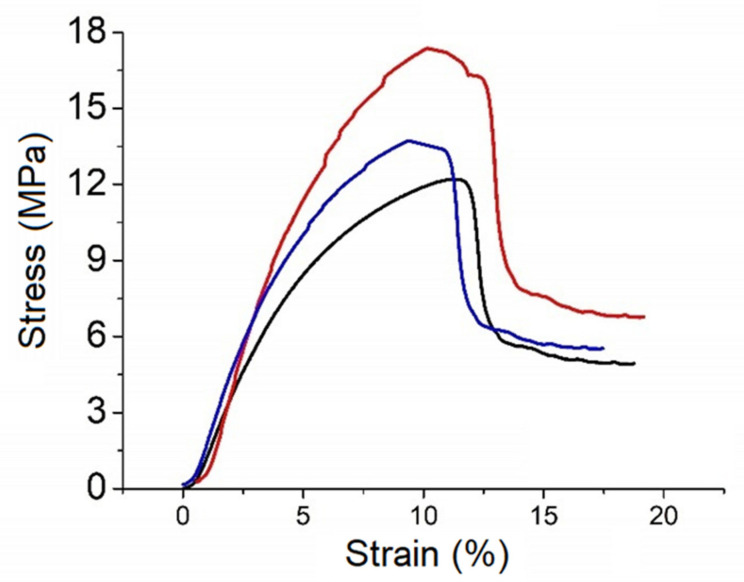
Stress-strain curves of three samples.

Figure 6 shows the stress-strain curves of 3 samples. The load was measured during the test and the data were converted into maximum engineering stress (*σ*_max_) and maximum engineering strain (*ε*_max_).

Table 4 shows the mean mechanical parameters of our samples and those of commercial collagen membranes for dental applications [19,20,21]. One can see that the mechanical parameters of collagen membranes show a large dispersion. This is attributed to differences of collagen types and manufacturing processes.

Sam et al. [22] evaluated the mechanical properties of platelet-rich fibrin (PRF) membrane, bovine collagen membrane and fish collagen membrane by performing surface indentation tests using a T1 950 Triboindenter (Hysitron Nanotechnology, Minneapolis, MN, USA). The Young modulus of elasticity was found to be 0.35 GPa for PRF membranes, 2.74 GPa for bovine collagen membranes, and 1.92 GPa for fish collagen. In the present work, the Young modulus of the non-crosslinked type 1 bovine derived collagen membrane calculated from the tension test was 3.58 GPa. The difference between the results found in this work and those reported in the literature is attributed to the measurement method.

### 3.4. DSC

The DSC technique is commonly used to assess the stability and thermal denaturation of collagen and its derivatives. In the present work, the critical temperatures of membrane collagen denaturation (*T*_onset_, *T*_endset_, *T*_p_) and the enthalpy of the endothermic reaction (Δ*H*) were measured.

**Figure 7:**
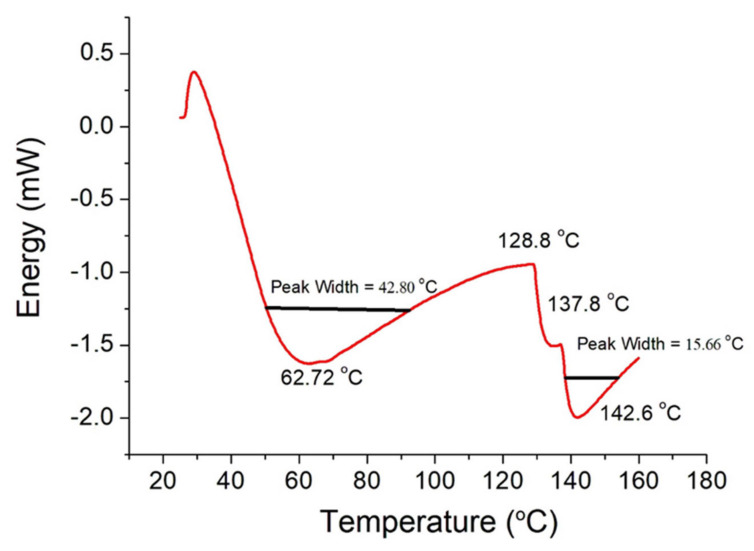
DSC thermogram of a sample.

Figure 7 displays the DSC thermograms of a resorbable collagen type I membrane. The denaturation temperature (*T*_d_) is defined as the temperature at which the greatest variety of energy in collagen heating occurs. A higher *T*_d_ reflects increased thermal stability of the collagen, while a broad shape of the peak and/or the presence of shoulders corresponds to increased dispersion in terms of stability of the fibers.

The DSC curve (Figure 7) shows two endothermic events. The first event is close to 60 °C and is related to the glass transition of a disorganized collagen fraction. The second event, located near 140 °C, is related to the loss of water. Literature results show a third event at 230 °C, which is related to denaturation of the chains of collagen molecules. The third event was not detected in the present work because measurements were taken only up to 150 °C. The phenomenon of denaturation of collagen type I began (*T*_onset_) at 41.3 °C, the peak temperature (*T*_p_) occurred at 62.72 °C, and the completion temperature (*T*_endset_) was 84.1 °C. The reaction enthalpy was 42.1 J/g.

The denaturation temperatures measured in the present work were different from those reported in the literature (Table 4). The transition temperature and energy of collagen are different among researchers. Differences in the temperature parameters (*T*_onset_, *T*_p_ and *T*_endset_) can be attributed to several factors, such as type of collagen (natural or synthetic), amino acid content, sample preparation, genetic line, animal age, fibrillation, mineralization, etc. Samouillan et al. [23] observed that the denaturation temperature of freeze-dried collagen type I is very distinct from that of the hydrated material. The number, arrangement, and thermal stability of the crosslinks are the most important factors in determining the denaturation temperature.

### 3.5. Immunohistochemistry

**Figure 8:**
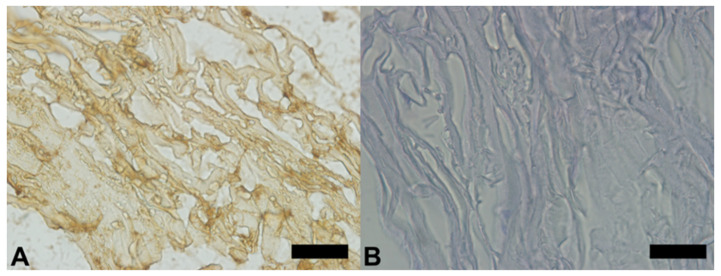
Morphology of membranes obtained in immunohistochemical analyses. (**A**) Type 1 collagen fibers extended throughout the sample, immunostained in brown, without the presence of other substances. (**B**) A negative control test, where there were no immune stains, certifying the efficiency of the process. Scale bar 100 µm, ×400 magnification.

The same pattern of type 1 collagen fibers identified in SEM was observed in the image obtained through immunohistochemical analysis (Figure 8). In addition, the immunohistochemical results show that there are no other substances in the samples.

## 4. Discussion

Roughness and wettability are complementary properties of type 1 collagen membranes and influence the tissue regeneration reactions. When the membrane has a porosity that allows the passage of nutrients and gases but does not allow the passage of aggressor cells, clinical results show results with bone growth and formation [24,25]. The membrane must have a roughness with micrometric dimensions to allow the adhesion and differentiation of osteoblastic cells. The adequate roughness to allow the adhesion of osteoblasts is around 1.2 µm [5], values similar to those of the membranes analyzed in the present work. The results presented in this work show that the roughness the membrane surface, together with adequate wettability, facilitates the tissue regeneration process.

Table 4 shows that membranes for dentistry applications have very different mechanical properties. To explain this behavior, it is important to analyze the raw material used to manufacture the membrane. Bio-Gide^®^ (Geistlich Biomaterials, Baden-Baden, Germany), Ossix Plus^®^ (Datum Dental Biotech, Lod, Israel), Collprotect^®^ (Botiss Biomaterials, Florence, Italy), and Jason^®^ (Botiss Biomaterials, Florence, Italy) are collagen membranes of natural origin (porcine). Bio-Gide is a bilayer membrane made of porcine dermis type I and III collagen. Ossix Plus is made from sugar-induced cross-linked collagen made of porcine tendon type I collagen. Collprotect^®^ membrane originates from the dermis, its structure is a dense network of collagen bundles with pores for better vascularization, as reported in the manufacturer’s datasheet. Remaix™ (RX, Matricel GmbH, Herzogenrath, Germany) is made from natural not-crosslinked collagen and contains elastin fibers. The membrane investigated in this work is a natural membrane made with bovine tendon type I collagen with highly organized fiber layers.

Ortolani et al. [20] analyzed the tensile properties of three commercially available collagen membranes of natural origin (Bio-Gide^®^—Geistlich Biomaterials, Baden-Baden, Germany; Collprotect^®^—Botiss Biomaterials, Italy, and Jason^®^—Botiss Biomaterials, Italy). They observed a bilinear stage in the first part of the curves up to the point where failure occurs. After fracture, they observed a residual strain because part of the membrane continues to stretch. According to Ortolani et al., stretching is probably one of the reasons for the initial bilinear stage.

Table 4 shows that the membrane thickness does not have a significant influence on the mechanical properties [26]. The membrane tested by Jason has a smaller thickness (0.20 mm) and a higher maximum tensile stress (13.0 MPa) than Bio-gide^®^, Remaix^®^, and OssixPlus^®^. The differences among the collagen membranes properties shown in Table 4 may be explained by differences in materials and microstructures. The samples studied in this work had a maximum tensile strength of 14.43 MPa, well above Bio-gide^®^ and other commercial products, indicating a better clinical performance.

According to Raz et al. [21], the Ossix Plus membrane is synthetic, and its production involves ribose-induced crosslinking that decreases the elasticity. That is why the natural collagen Bio-Gide^®^ membrane and the membrane investigated in this work have greater elasticity than the OssixPlus^®^ membrane. As the membrane elasticity increases, the ability to adapt to more irregular surfaces at the surgical site increases.

To explain the phenomena of collagen denaturation that occurs during heating, it is necessary to analyze its structure. The basic structure of the type I collagen molecule, known as tropocollagen, consists of three polypeptide chains (two α1 and one α2). The chains are coiled to form a right-handed triple superhelix. The three chains have the same repeating amino acids (Gly-Pro-Hyp) [27].

Collagen generally contains about 30% glycine (Gly), 12% proline (Pro), 11% alanine (Ala), 10% hydroxyproline (Hyp), 1% hydroxylysine (Hyl), and small amounts of other amino acids [26,27,28]. The thermal stability of collagen is related to the relative amount of the amino acids, mainly proline and hydroxyproline. Hydroxyproline and proline contribute to the stability of the triple helix through the hydrogen bonds between the α chains. The higher the percentage of these amino acids, the greater the stability of the helices and the higher the denaturation temperature of the collagen. When collagen denaturation occurs, a mixture of collagen types is formed with one, two, and three polypeptide chains.

The collagen molecules are polymerized through the formation of covalent crosslinks between polypeptide chains. As the collagen ages, crosslinks become increasingly stable. The energy required to unfold collagen molecules is measured by the denaturation enthalpy (Δ*H*). The temperature at which unfolding takes place is called denaturation temperature (*T*_p_). The unfolding of collagen strands involves two stages: separating the triple helices into single helices and then unfolding the individual helices into random coils. The first step in the unfolding of type I collagen helix involves the disruption of water bridges (hydrogen bonds) between the three polypeptide chains of the molecule. The second step involves breaking the intrahelical hydrogen bonds of the *α* chains. When collagen is heated, breaking of the intramolecular hydrogen bonds promotes shrinkage of the collagen fibers, followed by solubilization and gelatinization. The amount of water in the collagen has a significant influence on the Δ*H* and *T*_p_. The greater the amount of water in the collagen, the higher the enthalpy Δ*H* and the lower the temperature *T*_p_ [28].

Table 5 shows that the denaturation temperatures measured by León-Mancilla et al. [29] and Kopp et al. [26] are significantly greater than those measured in the present work. This difference can be explained by a higher number and stability of crosslinks between polypeptide chains. According to Kopp et al. [26], the high enthalpy value of 85 J/g is due to a large number of stable crosslinks.

The values of Tp and Δ*H* obtained in the present work are close to literature values for the thermal denaturation of collagen from epimysium (the connective tissue membrane that surrounds the muscle) and intramuscular collagen [26,28,29]. The denaturation temperature measured in the present work was *T*_p_ = 62.7 °C, and the denaturation enthalpy was Δ*H* = 42.1 J/g. According to Zhao et al. [30], the temperature of collagen denaturation in the skin of pigs and cattle is between 60 and 65 °C. Other studies mention collagen denaturation temperatures between 30 °C and 60 °C, depending on the origin and type of collagen, with lower temperatures for fish collagen and higher for those of bovine and porcine origin.

Samouillan et al. [23] describe an endothermic phenomenon for collagen and pericardium between 60 and 80 °C (Table 5) which was associated with collagen denaturation. León Mancilla et al. [29] observed the first endothermic peak of collagen scaffolds in the temperature range between 85 and 90 °C, corresponding to the dehydration process of surface water. The second endothermic peak of collagen scaffolds was between 275 and 325 °C and was related to the loss of hydrogen bonds, leading to protein denaturation. Rochdi et al. [14] studied tissue extracted from *postrigor longissimus dorsi* muscle taken from a 6-year-old cow. The tissue was dried in the air after four successive baths in acetone to remove fat. The results were *T*_p_ = 58.6–61.6 °C, and Δ*H* = 45.6–78.1 J/g.

Brum et al. [7] analyzed the cytotoxic, ultrastructural characteristics of a 1-mm thick non-reticulated collagen membrane indicated for guided bone regeneration surgery. The authors observed that the morphology of the membrane promotes an increase in the differentiation of osteocytes [7]. A systematic review and meta-analysis work on guided bone regeneration concluded that the association of the membrane improves the performance of the biomaterial and promotes an increase in the survival of implants [27]. These results are the main reasons for the development of new biomaterials, such as the membrane described in this work.

In a work that analyzed several characteristics of cross-linked and non-cross-linked collagen membranes, it could be observed that, in most cross-linked membranes, it was necessary to add some kind of external agent (ultraviolet radiation, for instance, or treatment with chemical solutions of genipin (Gp), glutaraldehyde, or 1-ethyl-3-(3-dimethylaminopropyl) carbodiimide hydrochloride (EDC). Frequently, these external agents caused tissue irritation and even reached que vascular system, since they were found in other parts of the body. One of the solutions found to avoid cross-linking was to increase the number of collagen layers [31]. In Figure 2 of this work, we can see not only 5 layers of collagen but a total thickness of 2 mm without cross-linking, indicating that its response in the process of tissue regeneration will be favorable.

## 5. Conclusions

According to the results of the present work, type 1 non-cross-linked collagen membranes of bovine origin have the following characteristics:

- The collagen fibers are structured in five layers.
- The elasticity is adequate for complex procedure (Young modulus = 3.58 GPa).
- It has good wettability (60s = 55.89o).

The methodology used in this study provides a good characterization of the physicochemical and ultrastructural properties of collagen membranes. The results showed that the bovine non-cross-linked type 1 collagen membrane has enough elasticity, ductility, and mechanical strength for use in tissue regeneration procedures.

## Figures and Tables

**Table 1:** Membrane roughness parameters.

Sample	Ra (µm)	Rsk (µm)	Rms (µm)	Rku (µm)	PV (µm)	Rpk (µm)	Rk (µm)	R3z (µm)
1	6.38	−0.05	8.01	3.12	67.16	8.19	19.79	45.84
2	7.56	−0.18	9.53	3.07	66.74	8.41	22.75	57.25
3	6.38	−0.38	7.84	2.98	70.08	5.58	23.01	62.86
4	4.87	−0.21	6.40	4.27	74.53	7.42	14.15	60.13
5	7.05	−0.30	8.84	3.31	94.27	8.10	22.20	79.77
6	7.06	−0.31	8.85	3.31	87.70	8.00	22.20	78.07
Mean	6.55	−0.24	8.24	3.34	76.75	7.62	20.68	63.98
StdDev	0.94	0.12	1.10	0.47	11.56	1.05	3.40	12.95

**Table 2:** Contact angle of a NaCl drop with the membrane as a function of time after drop deposition.

	Sample						
Time (s)	1	2	3	4	5	Mean	Standard Deviation
0	82.56°	80.26°	80.26°	77.35°	78.24°	79.73°	2.03°
15			64.50°	69.38°	64.20°	66.03°	2.91°
30			53.71°	65.89°	61.06°	60.22°	6.13°
45			49.96°	62.58°	60.51°	57.68°	6.77°
60			45.61°	58.92°	56.89°	55.89°	7.17°

**Table 3:** Drop parameters as functions of time after deposition.

Parameter	Time after Drop Deposition (s)				
	0	15	30	45	60
Contact Angle (degrees)	79.73	66.03	60.22	57.68	55.89
Wetting Tension (mN/m)	14.65	31.43	43.21	46.96	37.72
Base (mm)	1.44	1.36	1.2121	1.11	0.90
Base Area (mm^2^)	1.62	1.45	1.15	0.96	0.64
Height (mm)	0.61	0.52	0.43	0.39	0.23
Sessile Volume (µL)	0.75	0.65	0.54	0.49	0.34
Sessile Surface Area (mm^2^)	3.05	3.04	2.89	2.78	2.55

**Table 4:** Thickness (*e*), maximum tensile stress (*σ*_max_), and maximum tensile strain (*ε*_max_) of the membranes investigated in this work and of commercially available membranes.

		*e* (mm)	*σ*_max_ (MPa)	*ε*_max_ (%)
	This work	0.31	14.43	18.46
Bozkurt A et al. [19]	Bio-gide	0.48	12.3	4.7
Ortolani et al. [20]	Collprotect	0.28	13.1	16.3
	Jason	0.20	13.0	17.9
Raz et al. [21]	Remaix	0.29	10.4	7.01
	OssixPlus	0.26	5.13	6.0

**Table 5:** Comparison of critical temperatures and enthalpy of the endothermic reaction of our samples with some literature results.

	Parameter			
	*T*_onset_ (°C)	*T*_p_ (°C)	*T*_endset_ (°C)	Δ*H* (J/g)
This work	41.3	62.7	84.1	42.1
Samouillan [23]		65.1		58.5
León-Mancilla [29]	85		90.0	
Rochdi [28]		58.6–61.6		45.6–78.1
Kopp et al. [26]	75		150.0	85.0
Zhao [30]		60.0–65.0		
Nistor [24]		73.0		48.0
